# Diagnostic value of circulating lncRNA ANRIL and its correlation with coronary artery disease parameters

**DOI:** 10.1590/1414-431X20198309

**Published:** 2019-08-12

**Authors:** Yao Hu, Jing Hu

**Affiliations:** 1 Department of Cardiovascular Medicine, Jiangxi Provincial People's Hospital, Affiliated to Nanchang University, Nanchang, China; 2 Department of Cardiovascular Medicine, The First Hospital of Nanchang, Affiliated to Nanchang University, Nanchang, China

**Keywords:** LncRNA ANRIL, Coronary artery disease, Diagnosis, Disease severity, Inflammatory responses, Overall survival

## Abstract

This study aimed to detect the expression of the long non-coding RNA (lncRNA) antisense non-coding RNA in the INK4 locus (ANRIL) and evaluate its correlation with disease risk, stenosis degree, inflammation, as well as overall survival (OS) in coronary artery disease (CAD) patients. A total of 230 patients who underwent diagnostic coronary angiography were consecutively recruited and assigned to CAD group (n=125) or control group (n=105) according to presence or absence of CAD. Gensini score was calculated to assess the severity of coronary artery damage. Plasma samples were collected and the expression ANRIL was detected in all participants. High-sensitivity C-reactive protein (hs-CRP), erythrocyte sedimentation rate (ESR), and cytokines including tumor necrosis factor-α (TNF-α), interleukin (IL)-1β, IL-6, IL-8, IL-10, and IL-17 in CAD patients were measured and OS was calculated. The relative expression of ANRIL was higher in CAD patients compared to controls (P<0.001). Receiver operating characteristic disclosed that ANRIL could distinguish CAD patients from controls with an area under the curve of 0.789 (95%CI: 0.731–0.847). Spearman's rank correlation test revealed that expression of ANRIL was positively correlated with Gensini score (P=0.001), levels of hs-CRP (P=0.001), ESR (P=0.038), TNF-α (P=0.004), and IL-6 (P<0.001), while negatively correlated with IL-10 level (P=0.008) in CAD patients. Kaplan-Meier curve revealed that high expression of ANRIL was associated with shorter OS (P=0.013). In conclusion, circulating ANRIL presented a good diagnostic value for CAD, and its high expression was associated with increased stenosis degree, raised inflammation, and poor OS in CAD patients.

## Introduction

Cardiovascular disease (CVD) is the leading cause of death worldwide for the last 15 years, resulting in approximately 15 million deaths according to the World Health Organization 2015 statistical report, causing a huge social economic burden (1). As a main type of CVD, coronary artery disease (CAD) is one of the most common chronic inflammatory diseases and characterized by the remodeling and narrowing of coronary arteries that transport oxygen and blood to the heart (2). Although management of CAD has improved, the prevalence of CAD is still raising and the prognosis far from satisfactory. Therefore, additional and accurate biomarkers should be explored for predicting CAD risk and monitoring disease severity to promote early diagnosis and good prognosis in CAD patients.

Long non-coding RNAs (lncRNAs), defined as transcripts longer than 200 nucleotides with no protein coding capacity, account for more than 68% of human transcriptome and are involved in both transcriptional and post-transcriptional regulation (3–7). The lncRNA antisense noncoding RNA in the INK4 locus (ANRIL), also known as cyclin-dependent kinase inhibitor 2B antisense RNA (CDKN2BAS), is located on the 9p21.3 locus and involved in the process of development and progression of many chronic diseases including CVD, intracranial aneurysm, and periodontitis (8,9). However, limited studies investigate the association of lncRNA ANRIL expression with disease conditions and inflammatory responses in CAD patients. Therefore, the purpose of this study was to detect the lncRNA ANRIL expression and evaluate its correlation with disease risk, stenosis degree, inflammation, as well as overall survival (OS) in CAD patients.

## Material and Methods

### Participants

Two hundred and thirty patients who underwent diagnostic coronary angiography due to chest pain or symptoms of suspected CAD from March 2013 to February 2015 were consecutively recruited for this study. According to the presence or absence of CAD, participants were assigned to either CAD group or control group. CAD was defined as the presence of ≥50% of luminal stenosis in at least one major coronary vessel according to the coronary angiography, which was determined by the agreement of two independent and experienced interventional cardiologists. The exclusion criteria for all participants were as follows: 1) age younger than 18 years; 2) complicated with cardiomyopathy, congenital heart diseases, valvular diseases, or vasospastic angina; 3) history of coronary artery bypass graft surgery; 4) history of pulmonary embolism, tumors, serious infection, malignant hematological disease, autoimmune diseases, systematic inflammatory disease, and heart or renal dysfunction; 5) pregnant or lactating women. Control subjects with regional wall motion abnormalities or a history of CAD were also excluded. Finally, a total of 125 patients with CAD were allocated to the CAD group, and 105 patients without CAD were allocated to the control group.

### Ethics

This study was conducted in accordance with the Declaration of Helsinki and approved by the Institutional Review Board of The First Hospital of Nanchang (China). All participants provided a written informed consent before enrollment.

### Baseline data collection

Baseline characteristics of all participants were recorded in detail, including age, gender, body mass index (BMI), hypertension, diabetes, smoke, family history of CAD, triglyceride (TG), total cholesterol (TC), high-density lipoprotein cholesterol (HDL-C), and low-density lipoprotein cholesterol (LDL-C).

### Assessment of Gensini score

We used the Gensini score to assess the severity of coronary artery damage. Calculation of the Gensini score was initiated by giving a severity score to each coronary stenosis as follows: 1 point for ≤25% narrowing, 2 points for 26 to 50% narrowing, 4 points for 51 to 75% narrowing, 8 points for 76 to 90% narrowing, 16 points for 91 to 99% narrowing, and total occlusion was scored as 32 points. Then, this score was multiplied by a factor that showed the importance of the lesion localization in the coronary arterial system. For localization, scores were multiplied by 5 for the left main coronary artery, 2.5 for the proximal left anterior descending (LAD) and left circumflex (LCX) arteries, 1.5 for the mid segment LAD and LCX, 1 for the distal segment of LAD and LCX, first diagonal branch, first obtuse marginal branch, right coronary artery, posterior descending artery, and intermediate arteries, and 0.5 for the second diagonal and second obtuse marginal branches.

### Blood samples

Venous blood samples of 5 mL were collected from CAD patients and controls after 12-h fasting. Then, samples were centrifuged at 1627 g for 15 min at 4°C, and the plasma was separated and stored at –20°C until being further assayed.

### Measurement of the lncRNA ANRIL

Total RNA was extracted from plasma with the use of TRIzol reagent solution (Invitrogen, USA) and cDNA synthesis was performed using the transcription kit (Toyobo, Japan) according to the instructions of manufacturer. Then, real-time quantitative polymerase chain reaction (RT-qPCR) was performed to measure the relative expression of ANRIL with the use of SYBR Premix Ex Taq kit (Takara, Japan) and Applied Biosystems 7500 Fast Real-Time PCR system (USA). The relative expression of ANRIL was normalized to the expression of glyceraldehyde 3-phosphate dehydrogenase (GAPDH) and was calculated using the 2^−ΔΔCt^ method. The primers were as follows: ANRIL: forward: 5′-TGCTCTATCCGCCAATCAGG-3′, reverse: 5′-GGGCCTCAGTGGCACATACC-3′; GAPDH: forward: 5′-GAGTCAACGGATTTGGTCGT-3′, reverse: 5′-TTGATTTTGGAGGGATCTCG-3′.

### Measurement of inflammatory markers and cytokines

For CAD patients, the level of erythrocyte sedimentation rate (ESR) was measured by the PUC-2068A ESR analyzer (Perlong Medical, China). The plasma hs-CRP was analyzed using a particle-enhanced immunoturbidimetric method with the 7060 Automatic Biochemical analyzer (Hitachi Ltd., Japan). Concentrations of TNF-α, IL-1β, IL-6, IL-8, IL-10, and IL-17 were determined using the human enzyme linked immunosorbent assay (ELISA) kits (Abcam, USA) according to the instructions of the manufacturer.

### Follow-up

All CAD patients were followed-up regularly. The median follow-up duration was 42.0 months (range: 5.0–54.0 months). A total of 18 CAD patients were lost to follow-up until the last follow-up date of March 31, 2018 and were excluded from the OS analysis (from enrollment to the date of death from any cause).

### Statistical analysis

The sample size calculation was based on the pilot experiment, which included 20 CAD patients and 20 controls. In the pilot experiment, the ANRIL relative expression was 2.427±2.241 (means±SD) in CAD patients and 0.905±0.591 in controls. Using a two-sided *t*-test, 90% power to detect a difference in the ANRIL relative expression of 1.4, with a two-sided 5% level of significance (α), a sample size of 7 participants in each group was needed. Accounting for loss to follow-up and bias in patient selection, a total of 230 patients were enrolled in the present study. SPSS 21.0 statistical software (IBM, USA) and GraphPad Prism 6.01 software (GraphPad Software Inc., USA) were used for statistical analysis and chart making. Count data are reported as number and percentage; continuous data are reported as mean and SD if normally distributed and as median (25th–75th quartiles) if not normally distributed. Comparison between two groups was determined by the chi-squared test, *t*-test, or Wilcoxon rank-sum test. Receiver operating characteristic (ROC) curve was used to evaluate the ANRIL diagnostic value for CAD, Spearman's rank correlation test was used for correlation analysis, and the Kaplan-Meier method and log-rank test were used to determine the OS difference between ANRIL high-expression patients and low-expression patients. Statistical significance levels were all two-sided, and a P value <0.05 was considered significant.

## Results

### Baseline characteristics of CAD patients and controls

As listed in Table 1, the mean age of CAD patients and controls was 63.3±10.8 and 61.6±9.2 years, respectively, and there were 101 males and 24 females in the CAD patients group, as well as 83 males and 22 females in the control group. No difference was found in age (P=0.192), gender (P=0.741), and BMI (P=0.185) between the two groups. In addition, CAD patients had lower HDL-C level (P=0.005), higher LDL-C level (P=0.047), and advanced Gensini score (P<0.001) compared to controls, while there was no difference in other baseline characteristics between CAD patients and controls (all P>0.05). In addition, the median value for CAD patients of hs-CRP, ESR, TNF-α, IL-1β, IL-6, IL-8, IL-10, and IL-17 are shown in Table 1.

**Table 1:** Baseline characteristics of coronary artery disease (CAD) patients and controls.

Parameters	CAD patients (n=125)	Controls (n=105)	P value
Age (years)	63.3±10.8	61.6±9.2	0.192
Gender (Female/Male)	101/24	83/22	0.741
BMI (kg/m^2^)	23.8±2.5	23.3±3.1	0.185
Hypertension (n/%)	103 (82.4)	77 (73.3)	0.097
Diabetes (n/%)	25 (20.0)	17 (16.2)	0.456
Smoke (n/%)	57 (45.6)	49 (39.2)	0.872
Family history of CAD (n/%)	34 (27.2)	24 (22.9)	0.450
TG (mmol/L)	1.76±0.93	1.65±0.82	0.347
TC (mmol/L)	4.85±1.62	4.52±1.50	0.773
HDL-C (mmol/L)	1.10±0.34	1.22±0.31	0.005
LDL-C (mmol/L)	2.88±1.12	2.60±0.98	0.047
Gensini score	47.50 (26.00–72.75)	1.00 (1.00–2.00)	<0.001
hs-CRP (mg/L)	13.01 (18.09–29.38)		
ESR (mm/H)	26.52 (20.87–32.46)		
TNF-α (pg/mL)	44.93 (32.16–59.02)		
IL-1β (pg/mL)	4.29 (2.95–6.33)		
IL-6 (pg/mL)	36.74 (30.21–44.26)		
IL-8 (pg/mL)	46.84 (34.19-68.16)		
IL-10 (pg/mL)	22.50 (16.25–29.21)		
IL-17 (pg/mL)	63.09 (43.26–87.40)		

Data are reported as means±SD, median (25th–75th value), or count (percentage). P<0.05 was considered significant (*t*-test, Wilcoxon rank-sum test, or chi-squared test). BMI: body mass index; TG: triglyceride; TC: total cholesterol; HDL-C: fasting high-density lipoprotein cholesterol; LDL-C, fasting low-density lipoprotein cholesterol; hs-CRP: hypersensitive C-reactive protein; ESR: erythrocyte sedimentation rate; TNF-α: tumor necrosis factor α; IL: interleukin.

### ANRIL relative expression in CAD patients and controls

**Figure 1:**
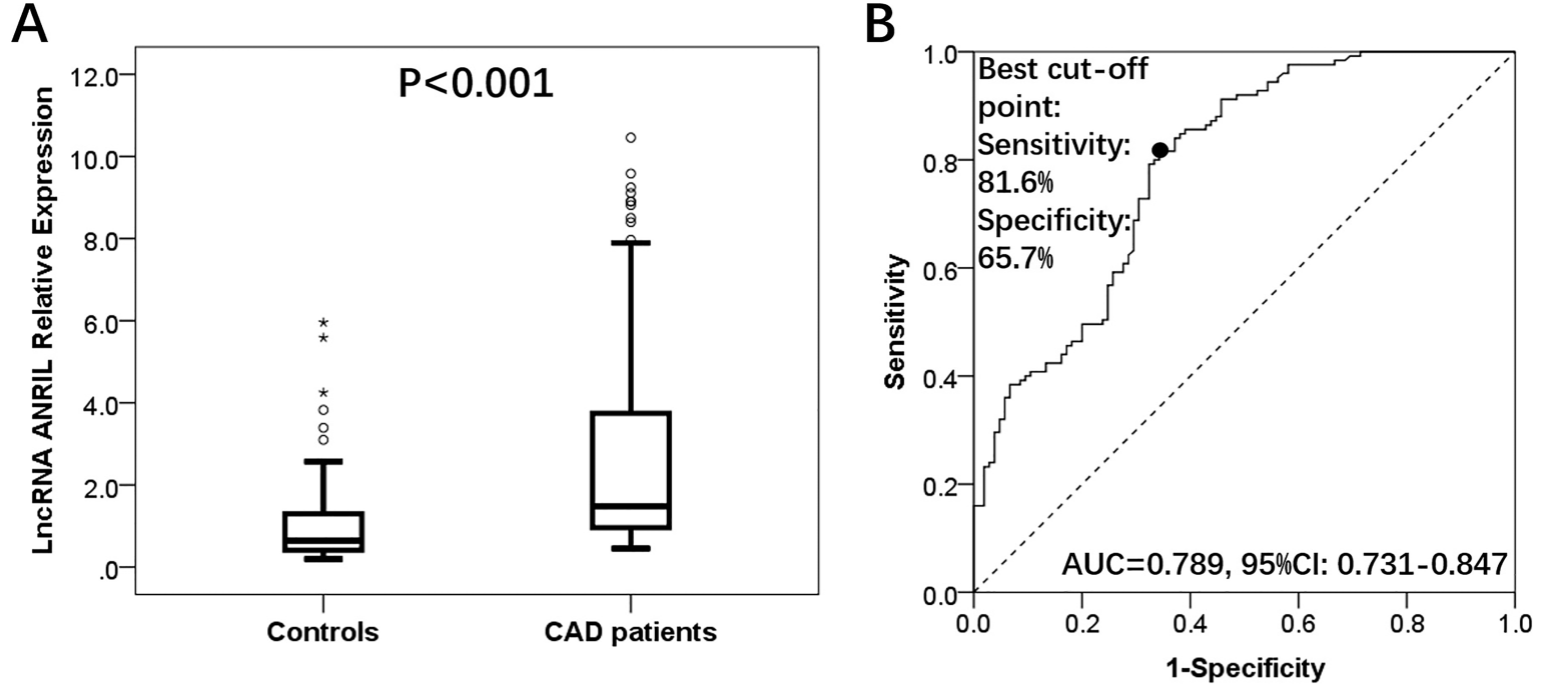
A, LncRNA ANRIL relative expression was higher in the CAD group compared to the control group. **B**, ANRIL could distinguish CAD patients from controls. Wilcoxon rank sum test was used to analyze the ANRIL relative expression between two groups. Receiver operating characteristic curve was used for the diagnostic value of ANRIL in CAD patients. Data are reported as median (25th–75th quartiles). P<0.05 was considered significant. LncRNA: long non-coding RNA; ANRIL: antisense non-coding RNA in the INK4 locus; AUC: area under the curve; CI: confidence interval; CAD: coronary artery disease.

The median value of ANRIL relative expression in CAD patients and controls was 1.481 (0.957–3.775) and 0.643 (0.409–1.388) (P<0.001; Figure 1A). ROC curve showed that ANRIL could distinguish CAD patients from controls with area under the curve (AUC) of 0.789 (95%CI: 0.731–0.847). Sensitivity and specificity were 81.6 and 65.7%, respectively, at the best cut-off point where the sum of sensitivity and specificity was the largest (Figure 1B).

### Correlation of ANRIL relative expression with Gensini score and inflammatory biomarkers in CAD patients

**Figure 2:**
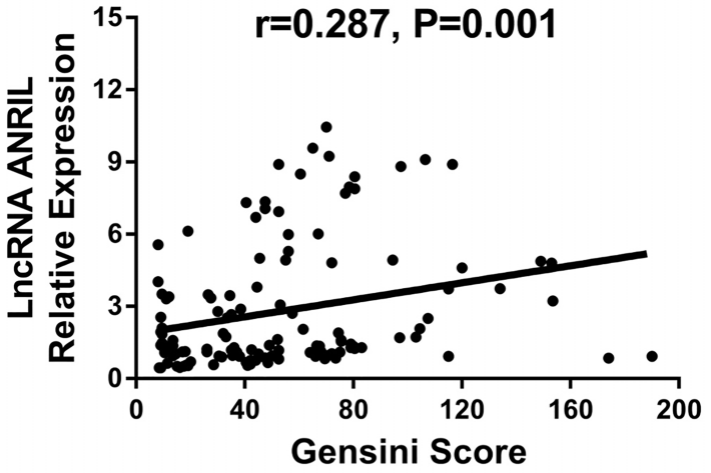
Correlation of lncRNA ANRIL relative expression with Gensini score in coronary artery disease patients. P<0.05 was considered significant (Spearman's rank correlation test). LncRNA: long non-coding RNA; ANRIL: antisense non-coding RNA in the INK4 locus.

**Figure 3:**
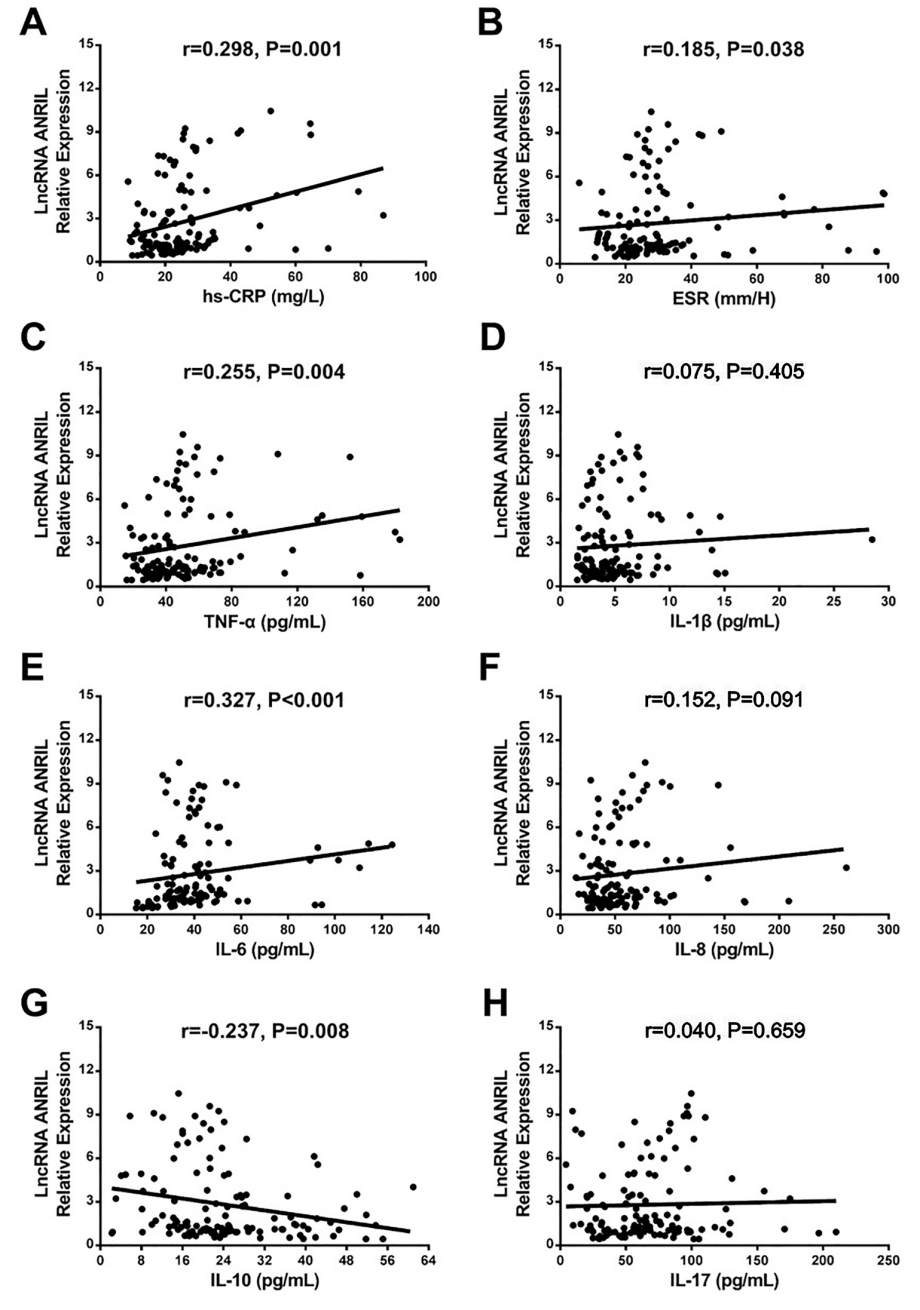
Correlation of ANRIL relative expression with inflammatory biomarkers in coronary artery disease patients: **A**, high-sensitivity C-reactive protein (hs-CRP); **B**, erythrocyte sedimentation rate (ESR); **C**, tumor necrosis factor-α (TNF-α); **D**, interleukin (IL)-1β; **E**, IL-6; **F**, IL-8; **G**, IL-10; and **H**, IL-17. P<0.05 was considered significant (Spearman's rank correlation test). LncRNA: long non-coding RNA; ANRIL: antisense non-coding RNA in the INK4 locus.

ANRIL relative expression was positively correlated with Gensini score (r=0.287, P=0.001; Figure 2). As for inflammation, ANRIL relative expression was positively associated with levels of hs-CRP (r=0.298, P=0.001; Figure 3A), ESR (r=0.185, P=0.038; Figure 3B), TNF-α (r=0.255, P=0.004; Figure 3C), and IL-6 (r=0.327, P<0.001; Figure 3E), while negatively correlated with IL-10 level (r=−0.237, P=0.008; Figure 3G) in CAD patients. No correlation was observed with IL-1β (r=0.075, P=0.405; Figure 3D), IL-8 (r=0.152, P<0.091; Figure 3F), and IL-17 (r=0.040, P=0.659; Figure 3H).

### Correlation of ANRIL relative expression and OS

**Figure 4:**
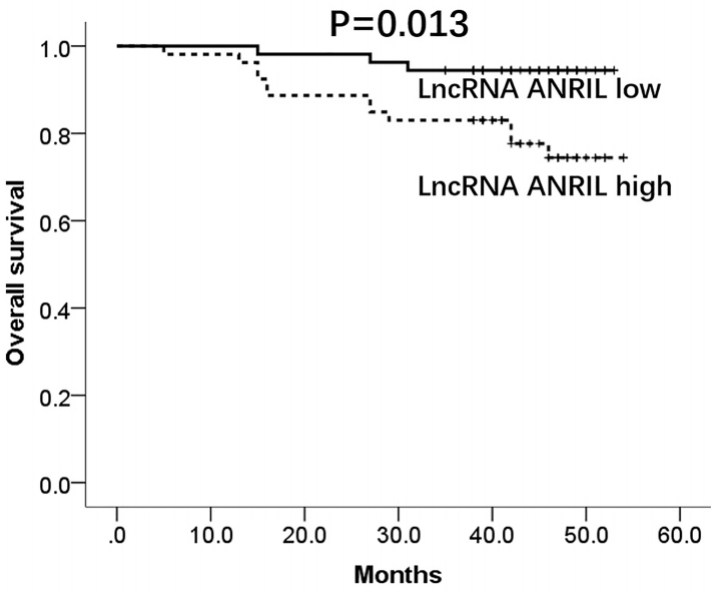
Comparison of overall survival between lncRNA ANRIL high-expression and low-expression coronary artery disease patients. P<0.05 was considered significant (Kaplan-Meier curve and the log-rank test). LncRNA: long non-coding RNA; ANRIL: antisense non-coding RNA in the INK4 locus.

According to medium value of ANRIL expression at 1.48, all patients were divided into high- and low-expression groups. The mean OS was 47.0 months (95%CI: 43.3–50.7) in the high-expression group and 51.4 months (95%CI: 49.6–53.2) in the low-expression group. Moreover, high expression was correlated with shorter OS in CAD patients (P=0.013; Figure 4). In addition, further multivariate Cox's regression model analysis was carried out to assess factors affecting OS, which disclosed that ANRIL relative expression (P=0.011, HR 1.763, 95%CI 1.138–2.732), hypertension (P=0.011, HR 92.352, 95%CI 2.782–3065.211), diabetes (P=0.008, HR 92.352, 95%CI 2.782–3065.211), and smoking (P=0.011, HR 50.573, 95%CI 2.501–1022.691) independently predicted poor survival in CAD patients (Supplementary Table S1).

## Discussion

In this study, we observed that: 1) ANRIL relative expression was higher in CAD patients compared to controls, it could distinguish CAD patients from controls, and it was positively associated with severity of coronary artery damage and systemic inflammation; 2) ANRIL high expression was correlated with worse OS in CAD patients.

Accumulating studies indicate that ANRIL is involved in the process of development and progression of CVD (2,10–14). For instance, ANRIL promotes expression of methylthioadenosine phosphorylase (MTAP) (one of atherosclerotic lesion compositions) to induce thrombogenesis and damage plaque stability, thereby accelerating the atherosclerotic process (13). ANRIL expression is also positively associated with p15^INK4b^ methylation, which contributes to CAD development (12). Moreover, ANRIL promotes inflammatory responses through activating NF-κB pathway and binding with Yin Yang 1 (YY1) in endothelial cells as well as cerebral infarction rat models (2,14). Taken together, these previous studies suggest that ANRIL might have an unfavorable role in development and progression of CAD.

Growing evidence indicates that ANRIL is highly expressed and associated with disease conditions in CVD patients (15-18). For example, ANRIL expression is elevated and positively correlated with numbers of plaque in atherosclerosis patients (15). Also, its high expression is associated with increased pro-inflammatory cytokines IL-6 and IL-8 levels in CAD patients (18). Although these previous studies indicated the unfavorable role of ANRIL in CVD patients, its potential value in diagnosis and correlation with disease conditions in CAD patients has not been clarified yet. The possible explanations might be: 1) ANRIL upregulated transcription of CAD-related protein coding genes including p15^INK4b^ and p16^INK4a^ to increase proliferation and migration in arterial smooth muscle cells and vascular smooth muscle cells, thereby contributing to high risk of CAD and increased stenosis degree of coronary artery; 2) ANRIL induced endothelial dysfunction via accelerating several signaling pathways such as TNF-α-NF-kB-ANRIL and YY1-IL6/8 pathways, thereby inducing inflammatory responses in pathological processes of CAD. However, the correlation between ANRIL relative expression and Gensini score as well as inflammatory biomarkers was relatively weak in this study, partly due to existing extrema.

The prognostic value of ANRIL has been elucidated in stroke and different types of cancers. A recent study indicates that its increased expression is correlated with high risk of recurrence in stroke patients (19). Another study reports that high ANRIL expression is associated with worse progression-free survival in multiple myeloma patients (20). In addition, ANRIL is an independent predictor for shorter OS and disease-free survival in nasopharyngeal carcinoma patients (21). As to the prognostic value of ANRIL in CAD patients, few studies are reported, and for the first time, we observed that its high expression was associated with shorter OS in CAD patients. This might result from: 1) ANRIL participated in the transcription process of protein coding genes on 9p21.3 locus (such p15 ^INK4b^ and p16^INK4a^ ) to increase cardiovascular cells proliferation and migration, thereby advancing CAD conditions and leading to unfavorable OS in CAD patients; 2) ANRIL aggravated inflammatory responses through stimulating several pathways including TNF-α-NF-kB-ANRIL and Yin Yang 1 (YY1)-IL6/8 pathways, leading to progressive inflammatory responses and poor OS in CAD patients.

Some limitations occurred in the present study. Firstly, most patients enrolled in this study were female; thus, further study with the same number of males and females is necessary. Secondly, all patients in this study were from only our hospital; hence, a multicenter study is necessary. Thirdly, follow-up duration of 42 months in this study was relatively short; thus, further studies investigating long-term effects of ANRIL on prognosis are needed in CAD patients.

In conclusion, circulating ANRIL had a good diagnostic value for CAD, and its high expression was associated with increased stenosis degree, raised inflammation, and poor OS in CAD patients.

## Supplementary Material

Click here to view [pdf].
